# Aortoiliac fenestrated stent demonstrates superior flow dynamics to kissing iliac stents in a large animal

**DOI:** 10.1016/j.jvssci.2026.100439

**Published:** 2026-08-17

**Authors:** Maxwell C. Braasch, Ryan Wahidi, Sophia R. Pyeatte, Santiago Elizondo-Benedetto, Mohamed Zaghloul, Hanxun Jin, John Cashin, DeVaughn Rucker, Shahab Hafezi, Batool Arif, Guy M. Genin, Mohamed A. Zayed

**Affiliations:** a Division of Vascular Surgery, Department of Surgery, Washington University in St Louis School of Medicine, St Louis, MO; b Cardiovascular Research Innovation in Surgery and Engineering Center, Washington University in St Louis School of Medicine, St Louis, MO; c Department of Biomedical Engineering, Washington University in St Louis McKelvey School of Engineering, St Louis, MO; d NSF Science and Technology Center for Engineering Mechanobiology, Washington University in St Louis McKelvey School of Engineering, St Louis, MO; e Division of Molecular Cell Biology, Washington University in St Louis School of Medicine, St Louis, MO; f Division of Surgical Sciences, Department of Surgery, Washington University in St Louis School of Medicine, St Louis, MO

**Keywords:** Aortoiliac bifurcation, Aortoiliac fenestrated stent, Endovascular treatment, Kissing iliac stents, Large animal

## Abstract

**Objective:**

Current endovascular treatment of aortoiliac occlusive disease with kissing iliac stents has suboptimal long-term patency and durability secondary to pathologic flow fields. The aortoiliac fenestrated (AIFEN) stent was previously introduced to provide an anatomically preserving endovascular therapy that may improve flow dynamics compared with kissing iliac stents. The aim of this study was to compare flow dynamics between the AIFEN stent and conventional kissing iliac stents in vivo in an adult porcine model.

**Methods:**

AIFEN stents were manufactured via benchtop modification of standard balloon-expandable covered stents. Three adult Yorkshire pigs underwent endovascular AIFEN implantation in the distal aorta with bilateral iliac stents (group 1), whereas three pigs received conventional kissing iliac stents (group 2). Over 4 weeks of survival, patency was assessed using weekly femoral ultrasound. Pre-explant computed tomography angiography with computational flow modeling was used to evaluate arterial flow dynamics, including vorticity and flow rate.

**Results:**

All pigs in both groups survived the 4-week study period without complications. Fluoroscopy at implantation and terminal procedures confirmed patent stents in all animals. Serial ultrasound demonstrated patent bilateral femoral artery outflow throughout the study. Computed tomography angiography-based flow dynamic analysis revealed that group 1 had significantly reduced iliac artery vorticity (*P* = .02) and higher flow rates (*P* = .004) compared with group 2.

**Conclusions:**

The anatomically designed AIFEN stent demonstrated superior flow dynamics compared with kissing iliac stents in a porcine model. These findings support further development of anatomically preserving balloon-expandable stent designs to improve endovascular treatment of aortoiliac occlusive disease.

**Clinical relevance:**

Endovascular treatment of aortoiliac occlusive disease commonly relies on kissing iliac stents; yet, this configuration alters native aortoiliac geometry and can generate disturbed flow patterns that contribute to thrombosis. In this large-animal study, an anatomically preserving aortoiliac fenestrated stent configuration demonstrated significantly improved flow dynamics compared with conventional kissing iliac stents, including reduced vorticity and increased flow rates. By maintaining more physiologic aortoiliac architecture, the aortoiliac fenestrated approach may mitigate adverse hemodynamic conditions associated with current endovascular strategies. These findings support further development of anatomically guided stent designs for the infrarenal aortic bifurcation that are aimed at improving durability and outcomes in patients with aortoiliac occlusive disease.

**Article Highlights**

• **Type of Research:** Large animal model
• **Key Findings:** The anatomically designed aortoiliac fenestrated stent demonstrated superior flow dynamics compared with kissing iliac stents in a porcine model.
• **Take Home Message:** These findings support further development of anatomically preserving balloon-expandable stent designs to improve endovascular treatment of aortoiliac occlusive disease.

Peripheral arterial occlusive disease is morbid and is associated with a significant health burden,^1^ occurring in 15% to 25% of adults over 70 years of age.^2^, ^3^, ^4^ Chronic aortoiliac occlusive disease is characterized by obstructions involving the distal abdominal aorta and proximal iliac arteries. Intervention is often performed to restore lower extremity arterial inflow. This is performed in two ways: endovascular stenting or open arterial bypass. While endovascular stenting is associated with lower short-term morbidity,^5^, ^6^, ^7^, ^8^, ^9^ open arterial bypass has higher long-term patency.^10^, ^11^, ^12^

Endovascular management of chronic aortoiliac occlusive disease is often performed by placing iliac artery stents that extend into the distal aorta. The side-by-side configuration that raises the native aortic bifurcation of the stents is often referred to as “kissing” iliac stents. In this procedure, two balloon-expandable covered stents are deployed across the distal aorta and bilateral common iliac arteries (CIAs). Pathologic flow fields through nonanatomic kissing iliac stents may contribute to limited long-term patency.^5^^,^^8^^,^^9^^,^^13^

Our group previously introduced a more anatomical aortoiliac fenestrated (AIFEN) stent configuration to improve the long-term patency of endovascular management of aortoiliac occlusive disease.^8^ A proof-of-concept first-generation AIFEN was shown to have feasible percutaneous endovascular implantation.^9^ Given the more anatomically configured AIFEN, we hypothesized that it would have a favorable impact on arterial patency and inflow dynamics in the aortoiliac segment compared with the standard-of-care kissing iliac stents. Here, we report a double-arm study comparison between AIFEN and conventional kissing iliac artery stenting in adult Yorkshire pigs.

## Methods

### Stent construction

**Fig 1:**
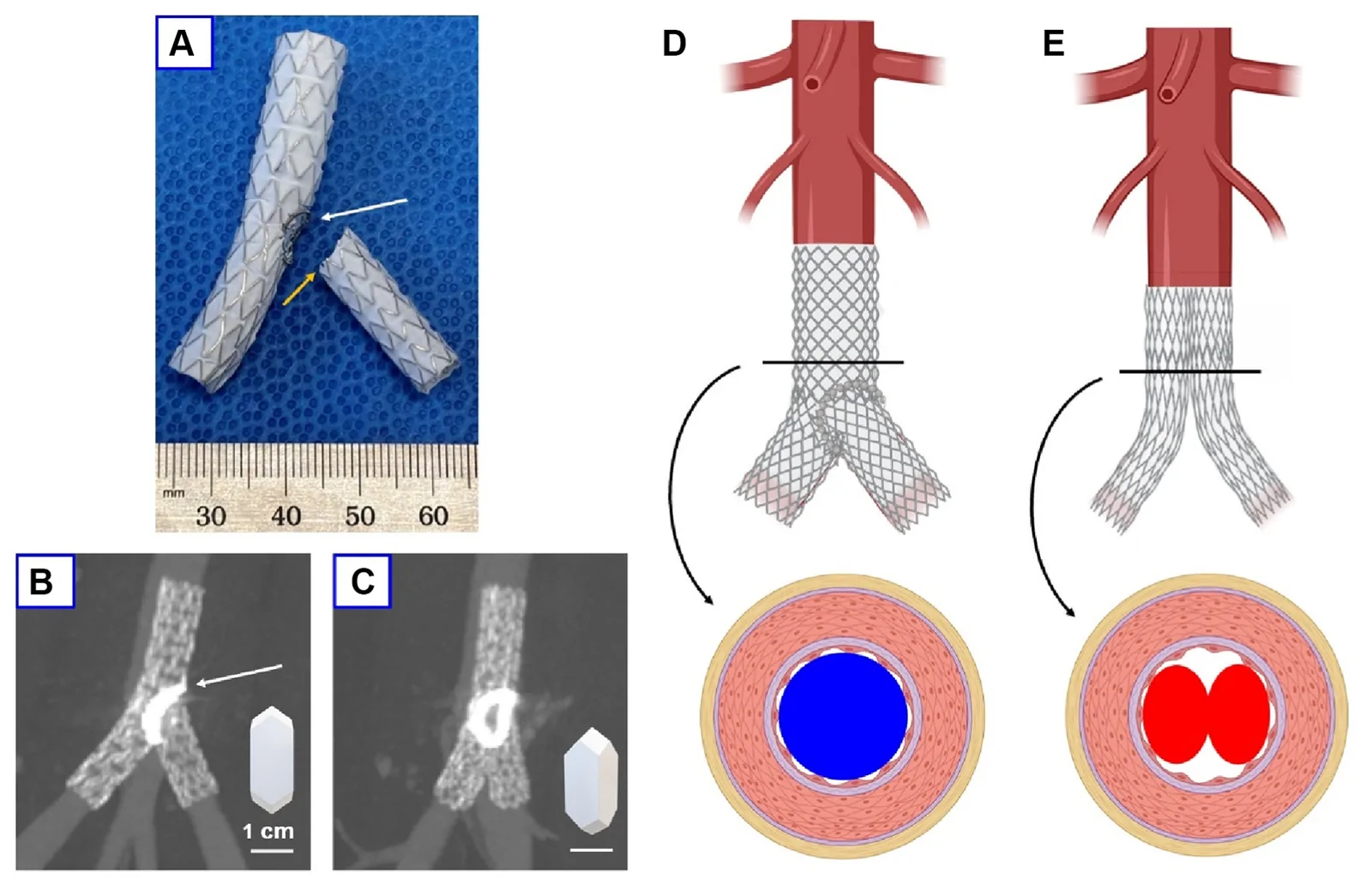
Design of the aortoiliac fenestrated (AIFEN) stent. **A,** Benchtop construction of the AIFEN stent with the embolization coil sutured around the fenestration (*white arrow*) and the modified tapered end of the contralateral common iliac artery (CIA) stent (*yellow arrow*). **B,** (*white arrow* indicates fenestration) and **C,** Three-dimensional (3D) reconstruction of the AIFEN and modified left CIA stents in a porcine model with full coronal and left lateral views with 3D graphics for orientation. **D,** Diagram of the AIFEN and modified left CIA stents with a visual representation of the cross-section of the stent in the distal abdominal aorta in *blue*. **E,** Diagram of the kissing iliac stents with a visual representation of the cross-section of the stent in the distal abdominal aorta in *red*.

Three AIFENs were constructed via benchtop modification of 9.8 mm × 55.4 mm BD Lifestream Balloon-Expandable Covered Stents (Becton, Dickinson and Company) as previously described (Fig 1).^9^

Two modifications were made to the AIFEN. First, a radiopaque marker of the stent fenestration was accomplished by suturing an 18 S 7/3 Tornado Embolization Microcoil (COOK Medical) around the fenestration using a running 6-0 monofilament suture (Fig 1, *A-C*). Prior to suturing the embolization coil to the fenestration, the prothrombotic fibered material was removed from the coil. Second, contralateral iliac stents were modified by transecting the proximal edge of 6.9 mm × 24.5 mm BD Lifestream Balloon-Expandable Covered Stents across the length of the distal strut for optimal contact with the AIFEN stent fenestration.

For kissing iliac stent endovascular implantations, unmodified 7.9 mm × 34.5 mm BD Lifestream Balloon-Expandable Covered Stents were utilized. The difference in stent configuration between AIFEN and kissing iliac stents in the distal abdominal aorta is demonstrated (Fig 1, *D* and *E*).

### Animals

All pig procedures were conducted using a study protocol approved by the Washington University in St Louis School of Medicine Animal Studies Committee. Porcine care and experimentation were performed with ethical and humane considerations.^14^

A total of six adult male Yorkshire pigs weighing 66 to 73 kg were procured from a commercial vendor (Oak Hill Farm). Pigs were housed individually in separate pens and allowed to acclimate in our facility for 72 hours prior to the initial implantation procedure. Pigs were randomized to group 1 (AIFEN) or group 2 (kissing iliac stent), and a total of three pigs were used for each group. Pigs in both groups had similar median weights (72 kg: interquartile range [IQR], 69-74 vs 71 kg: IQR, 71-72; *P* > .99). All pigs received standard twice-daily feeds and animal enrichment activities. Prior to any anesthesia-necessitating procedure, the pigs were fasted for 16 hours.

### Stent placement procedure

Endovascular implantations were performed in a controlled operating room setting. Sedation was induced with 4 mg/kg of Telazol, 2 mg/kg of ketamine, and 2 mg/kg of xylazine. All pigs were secured in a supine position on the operating table. Pigs were maintained on mechanical ventilation via endotracheal intubation with isoflurane 1% to 3% in 100% oxygen. Continuous noninvasive monitoring of heart rate, respiratory rate, end-tidal CO_2_, and pulse oximetry was performed. Intraoperatively, pigs assigned to both groups were maintained at similar heart rate (92 ± 8.1 beats per minute vs 88 ± 11 beats per minute; *P* = .12) and temperature (average, 37 ± 0.70 °C vs 38 ± 0.58 °C; *P* = .70).

Ultrasound-based percutaneous arterial access of the bilateral femoral arteries at the groin crease was performed in all pigs, and systemic therapeutic anticoagulation was administered once during the procedure (100 U/kg of intravenous heparin). AIFEN in group 1 were deployed as previously described, with balloons expanded to nominal pressure (8 atm) for stent deployment, and postdilation of the AIFEN and contralateral CIA stent performed with a 10 mm and 7 mm Mustang balloon (Boston Scientific), respectively.^9^ The only change to the AIFEN procedure from the previous description was the use of a 9F Brite Tip (Cordis) sheath in the right common femoral artery (CFA) for placement of the AIFEN stent in place of a 12F Brite Tip sheath. Introduction of AIFEN through a lower profile sheath was accomplished via improved recrimping of the AIFEN stent after benchtop modification compared with prior description. For kissing iliac artery stenting in group 2, 7F sheaths were placed in the bilateral femoral arteries, and 0.035-inch stiff Amplatz wires (Boston Scientific) were advanced into the abdominal aorta. Over the endovascular wires, parallel balloon-expandable stents were positioned in the distal aortic bifurcation and into the proximal bilateral external iliac arteries. Balloons were expanded to nominal pressure (8 atm) for simultaneous stent deployment with subsequent postdilation with 8 mm Mustang balloons. Following completion angiography, wires and sheaths were withdrawn, and manual pressure was held on the puncture sites for 15 minutes for confirmed hemostasis. Pigs were given daily oral aspirin 325 mg and daily oral Plavix 75 mg until the terminal procedure. Analgesic medication was administered as needed to the pigs by veterinary staff upon emerging from anesthesia.

### Ultrasound monitoring and analysis

Group 1 and group 2 pigs underwent weekly serial duplex ultrasound evaluation of the arterial peak systolic velocity (PSV) in the bilateral femoral arteries at the groin level. The pigs were anesthetized in a controlled environment.

An internally developed, automated Python pipeline (Supplementary Methods, online only) was used to quantify PSV from the collected duplex ultrasound waveforms. For each cardiac cycle, the algorithm identified the topmost bright pixel along the spectral envelope and converted it to a physical velocity. The pipeline generated both visual and quantitative outputs: annotated images highlighting detected PSV points, and structured comma-separated value files containing pixel coordinates, calibrated PSV values, and cycle-wise summary statistics.

### Computed tomography angiography image acquisition

All pigs underwent computed tomography angiography (CTA) 3 days prior to the terminal procedure using a Siemens Biograph Vision 600 (Munich, Germany).^15^ Scans were acquired from the upper abdomen to the midthigh following intravenous administration of 100 mL iopamidol contrast (Isovue 370; Bracco Diagnostics). Images were acquired with the following parameters: 120 kilovoltage peak, 216 mA-seconds, 6 mm slice thickness, and a 512 × 512 pixel matrix.

### Computational flow modeling

CTAs were used to construct flow models using SimVascular vascular modeling and simulation software.^16^ Vascular lumens were segmented using semiautomated thresholding with manual refinement. Reduced-order models were generated using ROMSimulator within SimVascular, based on extracted vessel centerlines, diameters, and standardized segment lengths (6.5 cm proximal to the aortoiliac bifurcation and 7.5 cm distal to the bilateral external iliac artery origins).

Three-dimensional simulations were performed for the distal abdominal aorta (3 cm proximal to the bifurcation) and bilateral external iliac arteries (3 cm distal to the aortic bifurcation). Simulations assumed steady-state inflow conditions, rigid vessel walls, and a common resistance boundary condition (*R* = 400 MPa s/m^3^) using Navier-Stokes equations. Vorticity and flow rates were quantified using ParaView (Kitware).^17^ The primary outcome was iliac artery vorticity. All simulations were performed with antegrade flow direction only, mirroring antegrade flow observed in the in vivo porcine model.

### Terminal procedure

Group 1 and group 2 pigs were survived for 4 weeks following stent implants, and at the time of sacrifice, both groups had similar weights (median, 90 kg [IQR, 89-91] vs 89 kg [IQR, 88-90]; *P* = .50). Pigs were brought to the operating room, anesthetized, endotracheally intubated, positioned supine, and monitored similar to stent implantation. The final bilateral femoral artery duplex ultrasounds were obtained, and then an ultrasound-based percutaneous right femoral artery cannulation was performed. A 7F Brite Tip sheath was placed in the vessel over an endovascular wire, and an angiographic catheter was advanced into the aorta. An aortoiliac angiogram was then performed to evaluate stent patency.

Prior to sacrifice, midline laparotomies and right medial visceral rotations were used to expose the aortoiliac segments. Pigs were then euthanized with potassium chloride. After euthanasia was confirmed, the aortas were divided 1 cm cranial and 1 cm caudal to the implanted stents in the aortoiliac segments.

### Histopathologic analysis

After explant, the proximal 0.5 cm of aortic tissue through the distal 0.5 cm of left femoral tissue was removed and placed in 70% ethanol overnight for dehydration. Right femoral tissues were damaged on explant and unable to be analyzed. Each tissue sample was then embedded in paraffin, sectioned into three different 5 μm sections, and mounted on glass histology microscope slides. One section from each aortic segment 0.5 cm above the proximal edge of the implanted stents and one section from each left femoral artery segment 0.5 cm distal to the distal edge of the implanted stent was stained with hematoxylin and eosin, Verhoeff VanGieson (VVG), and Masson's trichrome to assess global tissue architecture, fibrosis, and intimal hyperplasia. Images were captured using the Hamamatsu NanoZoomer HT 2.0 microscope (Hamamatsu Photonics). The ratio of tunica intima to media thickness (intima-media thickness [IMT]) of the VVG images of both the left femoral artery and aorta was assessed by two independent, blinded experts at five different regions at ×20 magnification.

### Statistical analysis

Arterial PSV was evaluated weekly both as absolute values and as changes from the prior week. Outlier PSV values greater than 1 standard deviation (SD) above the average for all PSV values each week were removed, which resulted in 0 to 4 values removed for each PSV weekly reading. In the determination of changing PSV week to week, the 24 PSV values closest to the median of all values for each week were utilized for calculation of change because of heart rate variability. Comparisons using two-tailed Mann-Whitney *U* tests were performed to compare group 1 and group 2 PSV values.

Vorticity and flow volume were grouped into the aortic segment, combined right and left iliac artery segments, and reported as averages with SD. Individual left and right iliac artery comparison was not performed because of significant risk of type II error. Comparison of all fluid dynamic measurement types in the aorta and iliac artery segments was performed between group 1 and group 2 using Mann-Whitney *U* tests for nonparametric data and unpaired two-tailed Student *t*-tests for parametric data.

The IMT of the femoral and aortic tissue in group 1 and group 2 was reported as averages ± SD. Comparison between study groups was also performed using unpaired two-tailed Student *t*-tests.

## Results

### Stent placement

**Fig 2:**
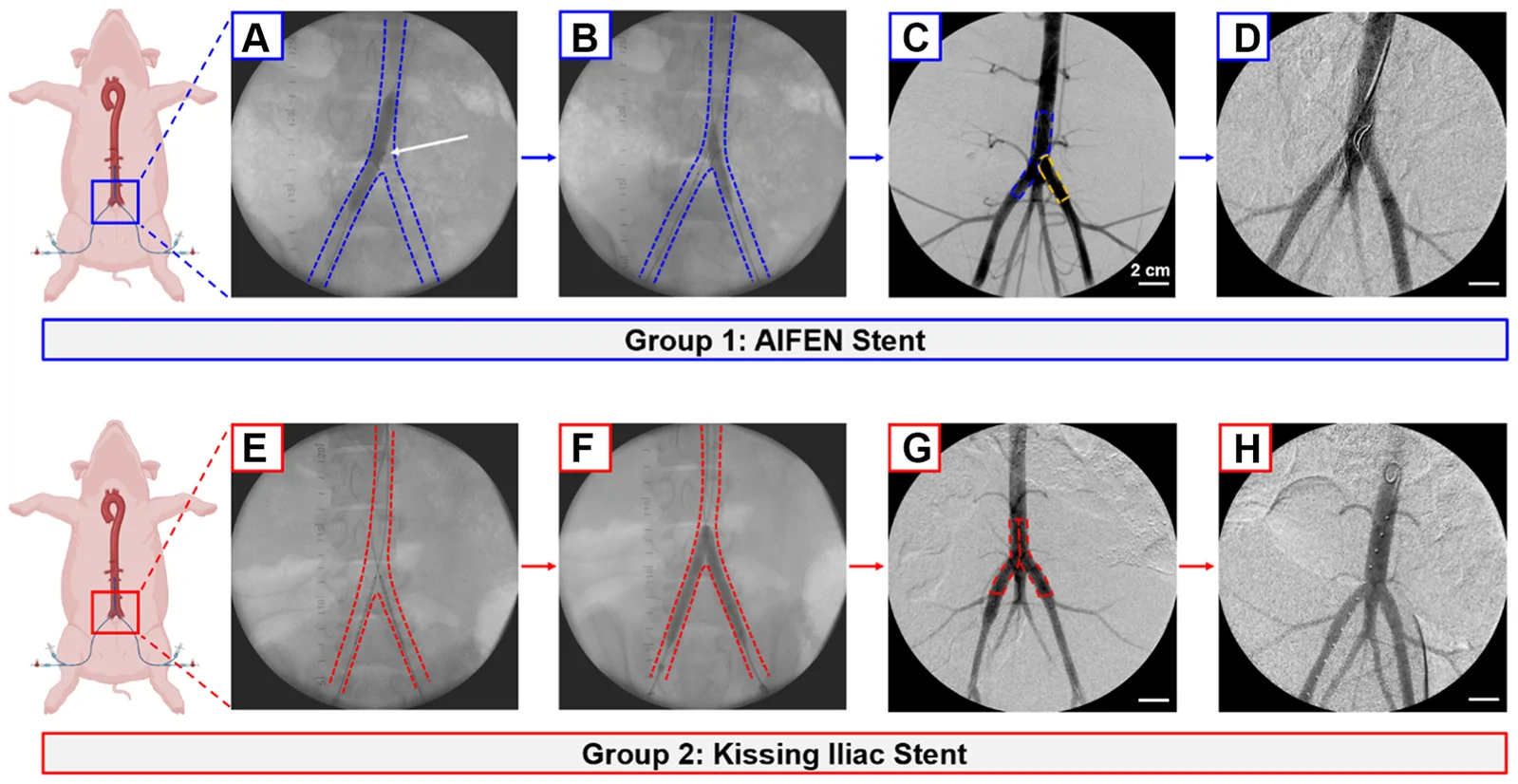
Placement of aortoiliac fenestrated (*AIFEN*) and kissing iliac stents in porcine models. **A,** Deployment of AIFEN with a contrast-filled noncompliant balloon in the distal abdominal aorta and the right common iliac artery (CIA) with the fenestration (*white arrow*) oriented toward the proximal left CIA. *Blue dashed lines* indicate the outline of the aorta and bilateral iliac arteries. **B,** Expansion of the modified contralateral stent in the left CIA abutting the fenestration of the AIFEN with guidewire placement through the fenestration. **C,** Angiogram immediately after AIFEN placement and deployment of the modified left CIA stent demonstrating complete patency of the aortoiliac system. **D,** Angiogram during the terminal procedure 4 weeks after AIFEN placement demonstrating complete patency of the aortoiliac system. **E,** Standard placement of kissing iliac stents just prior to expansion in the distal abdominal aorta and bilateral CIAs. *Red dashed lines* outline the aorta and bilateral iliac arteries. **F,** Simultaneous expansion of kissing iliac stents using contrast-filled noncompliant balloons by two different operators. **G,** Angiogram immediately after placement of the kissing iliac stents demonstrating complete patency of the aortoiliac system. **H,** Angiogram during the terminal procedure 4 weeks after kissing iliac stent placement demonstrating complete patency of the aortoiliac system. *AIFEN*, Aortoiliac fenestrated.

Group 1 pigs were successfully implanted with AIFEN and contralateral iliac artery stents (Fig 2, *A-D*). Placement of AIFEN was reproducible, and in all procedures, the fenestrations were easily orientable toward the contralateral CIA origin to facilitate placement of the adjacent iliac artery stent. The contralateral CIA stents were successfully placed with the proximal stent edge directly abutting the fenestration of AIFEN without overlap into the lumen of AIFEN. In group 2, kissing iliac stents were successfully deployed. Angiography immediately following stent implantations demonstrated fully patent aortoiliac systems in all six pigs with only antegrade flow through the aortoiliac systems (Fig 2, *E-H*). No intraoperative complications were encountered in either group.

### Survival period and terminal procedure

None of the pigs had groin hematomas, bleeding complications, puncture site infections, or abnormalities with gait or overall activity and behavior. Angiographic evaluation of the aortoiliac system at the terminal procedure 4 weeks following initial implantation demonstrated fully patent aortoiliac arterial systems in all pigs (Fig 2). CTA obtained prior to the terminal procedure facilitated three-dimensional reconstructions of the AIFEN implant (Supplementary Video 1, online only) and kissing iliac stents (Supplementary Video 2, online only). At explant of the stented aortoiliac systems in all pigs, no compression or collapse was observed in any portion of the AIFEN or kissing iliac stents. Similarly, no thrombotic material was observed in the AIFEN or kissing iliac stents.

### Ultrasound evaluation

**Fig 3:**
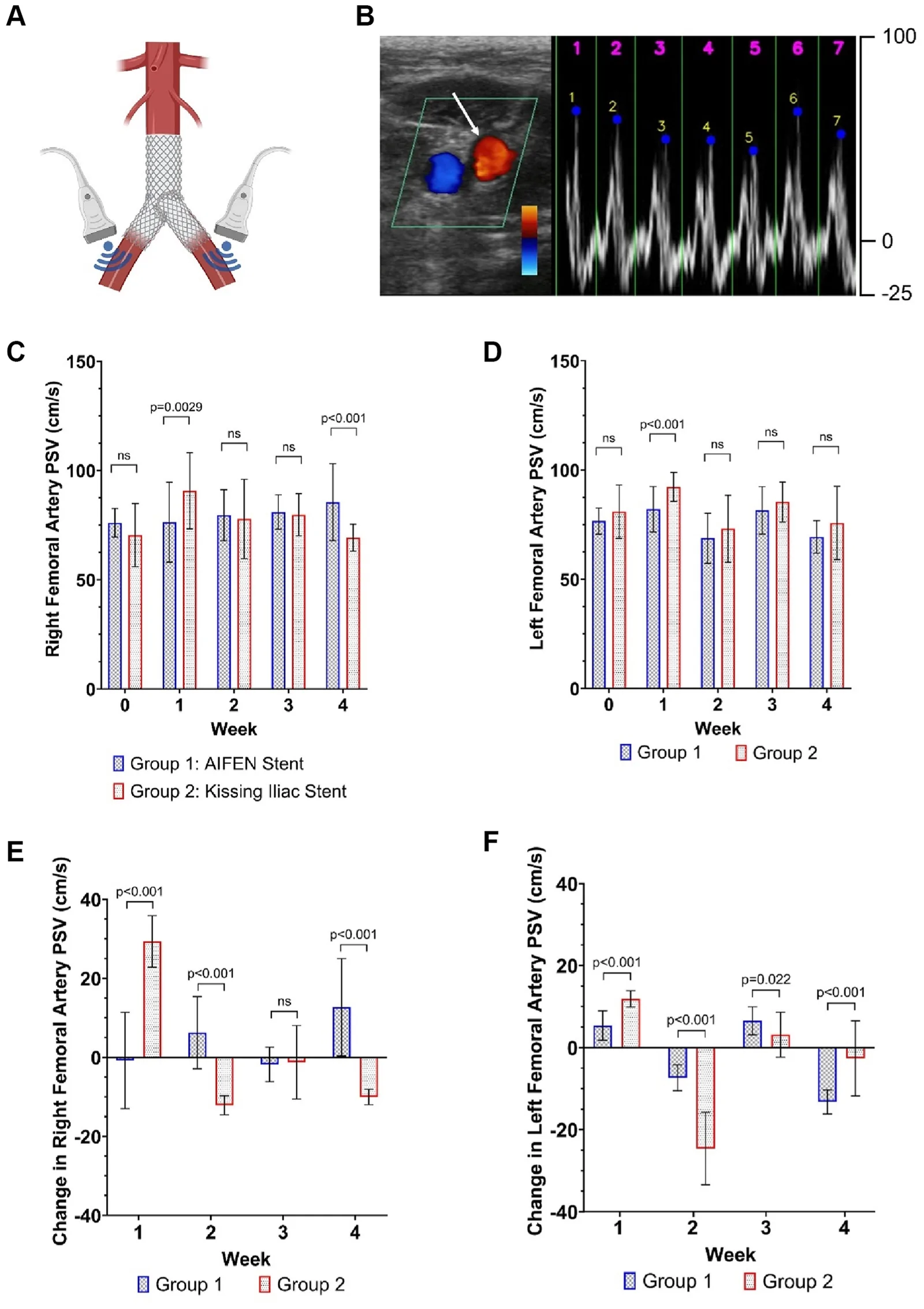
Duplex ultrasound of femoral artery outflow and PSV analysis over time. **A,** Diagram of ultrasound evaluation of the common femoral artery (CFA) distal to stents with a *white arrow* labeling the CFA. **B,** Representative image of an arterial duplex of the CFA distal to the aortoiliac fenestrated (AIFEN) stent with automated peak markings for PSV calculations (*blue dots* with *yellow numbers*) during each cardiac cycle (*purple numbers*). **C,** Weekly CFA PSVs of group 1 and group 2 in the right CFA. **D,** Weekly CFA PSVs of group 1 and group 2 in the left CFA. **E,** Weekly right CFA PSV changes from the prior week of group 1 and group 2. **F,** Weekly right CFA PSV changes from the prior week of group 1 and group 2. *PSV*, Peak systolic velocity.

Ultrasound evaluations demonstrated that all pigs had patent CFAs with only antegrade flow detected for each examination (Fig 3, *A* and *B*). Right femoral artery PSV was lower in group 1 at week 1 (median, 75 [IQR, 59-93] vs median, 84 [IQR, 74-108]; *P* = .029) and lower at week 4 (median, 70 [IQR, 66-73] vs median, 79 [IQR, 71-105]; *P* < .001) compared with group 2 (Fig 3, *C*). Left femoral artery values were also lower at week 1 in group 1 compared with group 2 (median, 82 [IQR, 75-88] vs median, 93 [IQR, 89-96]; *P* < .001; Fig 3, *D*).

In the right femoral artery, there was a significantly higher PSV change in group 2 compared with group 1 at week 1 (median, 25 [IQR, 25-37] vs median, −1.6 [IQR, −12 to 11]; *P* < .001), yet a lower PSV change in group 2 compared with group 1 at week 2 (median, −12 [IQR, −13 to −11] vs median, 5.5 [IQR, −2.5 to 15]; *P* < .001) and week 4 (median, −9.7 [IQR, −11 to −9.2] vs median, 7.2 [IQR, 1.3-24]; *P* < .001; Fig 3, *E*).

In the left femoral artery, there was a significantly higher PSV change in group 2 compared with group 1 at week 1 (median, 11 [IQR, 10-12] vs median, 5.6 [IQR, 1.9-7.9]; *P* < .001) and week 3 (median, 3.1 [IQR, −1.3 to 5.8] vs median, 7.1 [IQR, 6.0-8.9]; *P* = .22), yet a lower PSV change in group 2 compared with group 1 at week 2 (median, −23 [IQR, −29 to −17] vs median, −6.8 [IQR, −10 to −4.5]; *P* < .001) and week 4 (median, −3.9 [−4.9 to −0.16] vs median, −14 [IQR −15 to −12]; *P* < .001; Fig 3, *F*).

### Flow dynamic assessment

**Fig 4:**
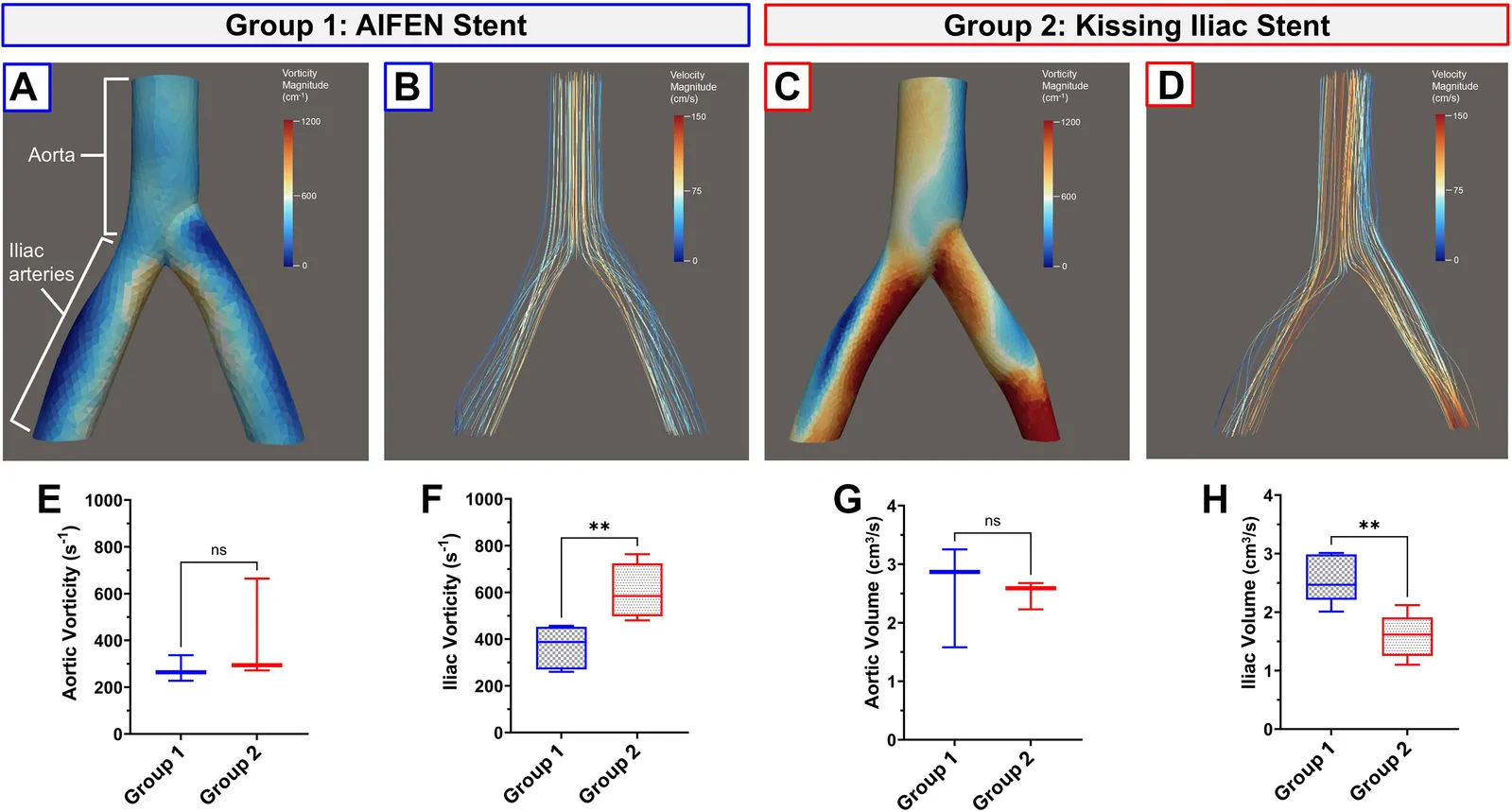
AIFEN demonstrates superior hemodynamic performance with reduced flow turbulence and enhanced iliac flow rates. Computational flow analysis from computed tomography angiography-based three-dimensional (3D) models at 4 weeks postimplantation. **A** and **B,** Vorticity heat maps reveal higher flow turbulence (*red regions*) in kissing iliac stents **(B)** compared with AIFEN **(A)**, particularly at the iliac origins. **C** and **D,** Velocity streamline visualizations demonstrate more laminar, organized flow through AIFEN **(C)** vs disturbed flow patterns in kissing iliac stents **(D)**. **E,** Aortic vorticity was comparable between groups. **F,** Iliac artery vorticity was significantly lower in AIFEN vs kissing stents (*P* = .02), indicating reduced flow turbulence. **G,** Aortic flow rates were similar between groups. **H,** Iliac artery flow rates were significantly higher in AIFEN vs kissing stents (*P* = .004), demonstrating improved hemodynamic efficiency of the anatomically preserving design. *AIFEN*, Aortoiliac fenestrated.

Computational flow analyses were performed on CTA-based three-dimensional models obtained at 4 weeks postimplantation in both group 1 and group 2 (Fig 4, *A-D*). Although vorticity in the aorta was similar between groups (Fig 4, *E*), vorticity in the bilateral iliac arteries in group 1 was lower than in group 2 (median, 387 [IQR, 301-436] vs median, 584 [IQR, 518-685]; *P* = .02; Fig 4, *F*). Flow volume in the aorta was similar between groups (Fig 4, *G*), but flow volume through the bilateral iliac arteries in group 1 was higher than in group 2 (median, 2.5 [IQR, 2.3-2.9] vs median, 1.6 [IQR, 1.3-1.8]; *P* = .004; Fig 4, *H*).

### Histopathologic evaluation

**Fig 5:**
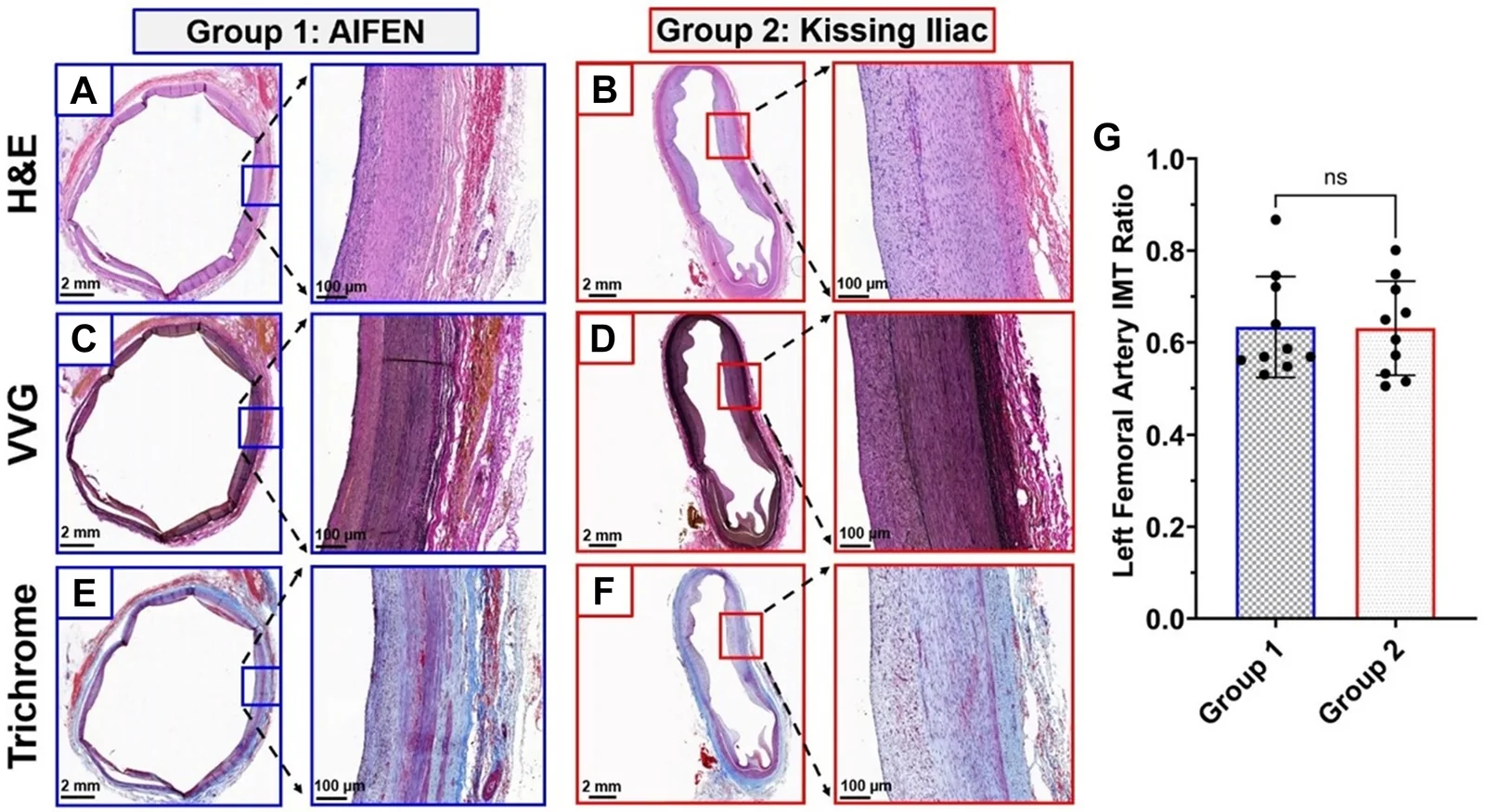
Histological evaluation of left femoral artery tissue distal to the AIFEN and kissing iliac stents. **A,** H&E staining of left femoral artery distal to the AIFEN stent in group 1 and **(B)** of left femoral artery distal to the kissing iliac stents in group 2. **C,** VVG section of the left femoral artery distal to the AIFEN stent in group 1 and **(D)** of left femoral artery distal to the kissing iliac stents in group 2. **E,** Trichrome staining of the left femoral artery distal to the AIFEN stent in group 1 and **(F)** of the left femoral artery distal to the kissing iliac stents in group 2. **G,** Aortic IMT was similar between group 1 and group 2 specimens. *AIFEN*, Aortoiliac fenestrated; *H&E*, hematoxylin and eosin; *IMT*, intima-media thickness; *VVG*, Verhoeff VanGieson.

Gross examination of hematoxylin and eosin, VVG, and Mason's Trichrome tissue cross-section staining of left femoral artery tissue demonstrated similar-appearing overall tissue architecture and elastin content (Fig 5, *A-F*). The left femoral artery tissue section IMT ratio was similar on VVG assessments between group 1 (average, 0.64 ± 0.11 vs 0.63 ± 0.10; *P* = .96). The aortic tissue section IMT ratio was also similar on VVG assessments between group 1 and group 2 (average, 0.091 ± 0.037 vs 0.071 ± 0.009; *P* = .11; Supplementary Fig, online only).

## Discussion

Our study demonstrates that when compared with kissing iliac artery stenting, in vivo implantation of the novel AIFEN presents multiple important advantages. First, the AIFEN benchtop modification and operative procedure for endovascular deployment were reproducible and reliable. Second, flow dynamic assessment demonstrated superiority of the AIFEN compared with the kissing iliac stent with regard to vorticity and total flow volume through the bilateral proximal iliac arteries at 4 weeks following stent implantation. These findings show the benefit of the AIFEN and highlight that AIFEN's superior flow dynamics may enhance durability and patency in the treatment of aortoiliac occlusive disease.

We previously reported the AIFEN design.^9^ Here, we provide a description of improvements to stent configuration, including improved radiopaque marking of the AIFEN fenestration and tapering of the proximal end of the contralateral iliac artery stent. These modifications improve technical endovascular maneuvering and positioning of the AIFEN main body stent as well as contralateral CIA stent placement. While no compression or collapse of the tapered proximal end of the contralateral CIA stent was observed, the longitudinal stability of this stent is unknown at this time. Since preoperative imaging was not available because of study budget limitations, stent sizes were estimated based on our prior knowledge of the Yorkshire adult aortoiliac anatomy and vessel diameters. In humans, preoperative CT assessments would allow for accurate stent size selections to appropriately accommodate human aortoiliac anatomy.^8^

The AIFEN and modified contralateral CIA stent can be effectively and safely deployed by a single operator. In comparison, kissing iliac stents require at least two operators to facilitate simultaneous stent deployment.^18^ Previously, the Covered Endovascular Reconstruction of the Aortic Bifurcation procedure was introduced to provide a more anatomic alternative to kissing iliac stents.^13^ However, two operators and three total stents are required, limiting Covered Endovascular Reconstruction of the Aortic Bifurcation integration into standard therapy. The AIFEN stent, in comparison, only requires two stents and one operator, making it less resource and time intensive. The AFX stent graft (Endologix) was previously introduced as a bifurcated self-expandable stent to treat aortoiliac aneurysmal disease^19^; however, difficulty in placement and reintervention limit its utility in wider clinical practice. The bifurcated in-stent in situ technique does permit stenting across bifurcations in patients with aneurysmal disease but still requires three total stents and creation of a fenestration after deployment that may not be feasible in larger aortoiliac anatomy.^20^ While the “Double D” technique may provide benefit to apposition of kissing iliac stents, overdilation may not be safely feasible in patients with significant aortoiliac atherosclerotic disease burden.^21^ The AIFEN stent is designed to provide inline flow through atherosclerotic segments of the aortoiliac system only. It is not designed to address aneurysmal disease. Similar to current standard practice, the covered stents of the AIFEN are intended to provide luminal seal in situations where arterial wall microfractures or ruptures occur.

Pigs implanted with the AIFEN demonstrated superior flow dynamics to pigs implanted with kissing iliac stents. High-shear environments are known to induce thrombus formation through platelet aggregation.^22^, ^23^, ^24^ Vorticity, a measure of the rotational component of fluid related to velocity and shear stress, is important in situations of steep stenotic geometry,^25^^,^^26^ such as aortic narrowing because of kissing iliac stents. Our analysis demonstrates higher vorticity in the proximal iliac arteries of pigs implanted with kissing iliac stents compared with the AIFEN. Higher vorticity induces platelet aggregation and subsequent thrombosis in the evaluation of extracorporeal membrane oxygenation,^27^ underscoring its criticality in thrombosis. Higher volume of arterial flow through the iliac arteries of the AIFEN compared with kissing iliac stents is another benefit that would help sustain arterial inflow in the setting of concomitant distal peripheral arterial occlusive disease.^28^ Visual representation demonstrated an acute increase in vorticity at the proximal end of the bilateral iliac arteries in the kissing iliac stent group. This acute increase was not observed in the AIFEN group. It is possible that this observation is secondary to the nonanatomic configuration of kissing iliac stents in the aorta, leading to a more abrupt increase in vorticity at the iliac bifurcation. Despite the strength of our computational flow model data and the clinical relevancy of vorticity to thrombosis, these data can only provide insight into long-term patency and flow of AIFEN and kissing iliac stents, not true observed long-term patency.

Results of both ultrasound and histological analysis support the benefit of the AIFEN. Duplex ultrasound monitoring of CFA flow distal to the stents is a reliable surrogate for assessment of proximal inflow arterial obstruction or in-stent stenosis.^29^ The rise in PSV at week 1 in group 2 suggests that its nonanatomical configuration is impacting arterial inflow—particularly since the more anatomical group 1 did not demonstrate a similar perturbation. Over subsequent weeks, it is possible that distal arterial collateralization in group 1 developed to correct for the initial arterial inflow perturbation. This may in part explain why greater changes in PSV values were observed in group 2 between weeks 2 to 3 and 3 to 4, when compared with group 1.

Greater arterial IMT has been linked to reduced arterial patency^30^, ^31^, ^32^ and a higher risk of distal thrombus formation.^24^ In our study, IMT did not differ between groups, and we observed no evidence of arterial stenosis or thrombosis. This lack of change is likely attributable to the relatively short postoperative surveillance interval of only 4 weeks.

### Limitations

We acknowledge some limitations in this study. First, the 4-week observation period precludes long-term observations of AIFEN patency relative to kissing iliac stents. However, the growth rate of Yorkshire pigs is rapid, between 1 and 1.5 lbs per day,^33^ which raises significant challenges with the longitudinal care and mobility of these animals. Femoral flow dynamic assessments were utilized to provide insight into the patency of the proximal aortoiliac stents and were highly feasible and reproducible. However, we acknowledge that these measurements may have led to missing subtle flow abnormalities in the iliac arteries. Longitudinal aortoiliac ultrasound via open laparotomy was not feasible in this study since it would require animal sacrifice. This limitation was mitigated with a completion angiogram at the terminal procedure. In future studies, animal sacrifice may be staggered to evaluate AIFEN patency longitudinally.

Our study was in Yorkshire pigs without aortoiliac atherosclerosis. We acknowledge that the presence of disease at the aortic bifurcation may make AIFEN deployment, particularly the contralateral CIA stent, more complicated. The balloon-expandable stent design is intended to facilitate radial fixation of both the main body AIFEN and contralateral CIA stent within the diseased lumen to avoid stent migration or bifurcation encroachment either in the acute setting or longitudinally with time. During our 4-week-long study, we did not observe any evidence of stent migration, and longer follow-up time intervals can provide further validation of this important observation.

It is possible that the presence of severe calcified atherosclerotic disease can also impact the AIFEN procedure's technical feasibility and success. Plaque fracture, dislodgement, and impact on alignment of the AIFEN fenestration will require further dedicated studies to confirm feasibility and safety. An additional anatomic consideration is the presence of a large sacral artery in Yorkshire pigs at the aortic bifurcation. While it is possible that this could have confounded our femoral artery flow results, it remained a consistent variable between animals studied.

Finally, our relatively small sample size of three pigs per group could have led to either type I or type II error. This limited our ability to analyze and compare vorticity and flow volumes through the left and right iliac arteries individually rather than in a grouped fashion. Nevertheless, the study findings provide clear differences in vorticity and flow rates between groups 1 and 2.

## Conclusions

The AIFEN stent was successfully implanted and remained patent for 4 weeks in a large animal Yorkshire pig aortoiliac segment. Compared with conventional kissing iliac stents, AIFEN demonstrated significantly reduced iliac artery vorticity and higher flow rates, indicating superior hemodynamic performance. These findings suggest that an anatomically preserving stent design may enhance long-term patency and durability for endovascular treatment of aortoiliac occlusive disease. Future verification and validation studies are needed to progress the AIFEN technology toward human clinical use.

## Author Contributions

Conception and design: MB, RW, SP, HJ, JC, DR, SH, GG, MZ

Analysis and interpretation: MB, RW, HJ, SH, BA, GG, MZ

Data collection: MB, RW, SP, SB, MZ, HJ, JC, SH, BA

Writing the article: MB, RW, HJ, MZ

Critical revision of the article: MB, RW, SP, SB, MZ, HJ, JC, DR, SH, BA, GG, MZ

Final approval of the article: MB, RW, SP, SB, MZ, HJ, JC, DR, SH, BA, GG, MZ

Statistical analysis: MB, RW, HJ

Obtained funding: GG, MZ

Overall responsibility: MZ

## Funding

This work received 10.13039/100000002National Institutes of Health funding (grant no. T32HL170959; principal investigator: Mohamed A. Zayed) that provided trainee support.

## Disclosures

M.A.Z. and G.M.G. are cofounders of Caeli Vascular, Inc and Vascorra LLC. Neither company is commercializing the endovascular technology reported in this manuscript, nor did they provide any financial assistance for this study. M.A.Z. is also a cofounder of AirSeal Cardiovascular, Inc, which is not commercializing a medical device. M.A.Z. is a consultant for Amgen, Medtronic, Ascera Surgical, and GlucoTrack. The remaining authors report no conflicts.
