# Supplementary material for: Aortoiliac fenestrated stent demonstrates superior flow dynamics to kissing iliac stents in a large animal
Source: JVS Vasc Sci. 2026 Aug 17;7:100439. doi: 10.1016/j.jvssci.2026.100439 (PMC13594900; doi:10.1016/j.jvssci.2026.100439)
Supplement: Supplementary file 2 [file mmc2.docx]

**Methods: Automated Analysis Pipeline for Ultrasound-Based Peak Systolic Velocity (PSV) Quantification**

We developed a Python-based image processing pipeline to quantify PSV from Doppler ultrasound images. The workflow integrates manual region-of-interest (ROI) selection with automated signal extraction, adaptive peak detection, velocity calibration, and annotated output generation to enable reproducible and efficient PSV analysis.

## Part 1: Image Preprocessing and ROI Selection

Raw Doppler ultrasound images, provided in .jpg format, often vary in resolution and aspect ratio depending on acquisition settings. To ensure consistency across samples and facilitate standardized processing, each image was first rescaled to a fixed target width of 1024 pixels using bicubic interpolation. This resizing step preserved the aspect ratio while enhancing the uniformity of spatial features across the dataset. Next, the user manually selected the ROI containing the spectral Doppler waveform. This interactive selection was implemented via OpenCV’s selectROI tool, which enabled precise control over the cropping boundaries by displaying the image in a GUI window with crosshairs and adjustable selection rectangles.

The selected ROI was assumed to include the full vertical range of the Doppler spectrum, bounded laterally by the start and end of the signal trace. This cropped region was saved separately and used as the input for all subsequent signal extraction and analysis procedures.

**Listing 1: Manual ROI Selection and Cropping**

This manual cropping step ensured that only the relevant Doppler signal was analyzed, avoiding artifacts or extraneous elements such as on-screen text, color bars, or surrounding tissue structures.

## Part 2: Signal Extraction and Peak Detection

To quantify PSV from Doppler ultrasound scans, we extract and analyze the intensity profile of the Doppler spectral trace. This section describes the signal extraction strategy, the mathematical formulation, and the adaptive thresholding logic critical for robust peak detection across variable-quality images. After manual cropping, each image was upscaled by a factor of two to enhance spatial resolution. The image was then converted to grayscale, and a brightness threshold was applied to eliminate low-intensity background noise. To convert the 2D Doppler spectrum into a 1D temporal signal profile, we computed the mean grayscale intensity along each image column:

$$s\left( x \right)=\frac{1}{H}\sum_{y=1}^{H} I\left( x,y \right), (S1)$$

where *s*(*x*) is the signal intensity at horizontal coordinate *x*, *I*(*x, y*) is the grayscale pixel value at location (*x, y*), and *H* is the image height. This signal *s*(*x*) effectively captures the envelope of the Doppler waveform, where peaks correspond to systolic velocity surges in each cardiac cycle. However, due to inter-sample variability and image noise, fixed thresholds fail to generalize. To address this, we used adaptive thresholding informed by the signal’s statistical properties:

$$P_{\mathrm{prom}}=\lambda_{p}\cdot\sigma_{s}, (S2)$$

$$P_{\mathrm{height}}=\mu_{s}+\lambda_{h}\cdot\sigma_{s}, (S3)$$

where *µ_s_* and *σ_s_* are the mean and standard deviation of the signal *s*(*x*), and *λ_p_*, *λ_h_* are user-defined scaling factors (typically *λ_p_* = 1*.*0, *λ_h_* = 0*.*3).

Prominence threshold ensures detected peaks are prominent relative to their surrounding valleys, filtering out noise-induced oscillations. Height threshold guarantees that only systolic events above baseline variability are captured. Peaks were then detected using SciPy’s find peaks()function, incorporating these adaptive thresholds and a minimum distance constraint between successive peaks to prevent over-segmentation:

**Listing 2: Adaptive Peak Detection**

## Part 3: Localization of Maximal Velocity Points

After detecting the peak positions that segment each cardiac cycle, the next step involves identifying the maximal velocity point within each heartbeat window. In Doppler ultrasound images, this corresponds to the top-most bright pixel within each segment, representing the point of highest flow velocity.

For each segment [*x_i_, x_i_*_+1_] defined by adjacent signal peaks, the algorithm scans each column *x* to locate the first pixel *y* from the top of the image that exceeds an intensity threshold *T*. Mathematically, the vertical position of the maximum velocity point within a segment is computed as:

$$y_{min}=\min\left\{ y | I\left( x,y \right)>T, x\in\left[ x_{i},x_{i+1} \right] \right\} (S4)$$

This procedure is repeated for all segments to produce a set of top-edge coordinates corresponding to the envelope of peak systolic velocities. A lower *y* value implies higher velocity in the image scale.

**Listing 3: Locating Maximum Velocity Points Per Segment**

## Part 4: Calibration of Y-axis and Velocity Conversion

To convert pixel-based vertical positions into physical units of velocity (e.g., cm/s), we employed a linear calibration procedure based on the Doppler scale bar displayed in each ultrasound image. The user was prompted to manually input the velocity values corresponding to the top (*y*_top_) and bottom (*y*_bottom_) of the Doppler spectral window, which are typically labeled on the y-axis of the ultrasound system.

Let *y_i_* denote the vertical pixel coordinate (in image space) of a detected maximum velocity point, and let *H* be the total height of the image. Because image coordinate systems typically assign the origin at the top-left corner (i.e., *y* = 0 at the top and *y* = *H* at the bottom), a higher velocity corresponds to a smaller *y_i_* value. Assuming a linear velocity gradient between *y*_top_ and *y*_bottom_, the velocity *v_i_* at a given pixel *y_i_* was computed using:

$$v_{i}=\left( 1-\frac{y_{i}}{H} \right)\left( y_{\mathrm{top}}-y_{\mathrm{bottom}} \right) (S5)$$

This transformation maps the pixel coordinate proportionally to the user-defined physical velocity range. This calibrated mapping is applied to all heartbeat cycles independently, enabling consistent comparison of PSV values across different scans and subjects. Importantly, this step relies on accurate user input for scale values and assumes that the Doppler spectral envelope is vertically linear and undistorted.

## Part 5: Output Generation and Annotation

To facilitate downstream analysis and visualization, the pipeline generates both visual and tabular outputs that summarize the detected peak systolic velocities for each cardiac cycle.

(1) **Labeled Image Output:** For each processed ultrasound scan, a labeled image is generated to visually confirm the detection results. The following annotations are overlaid on the upscaled and cropped grayscale image:

- **Red circles** mark the pixel locations corresponding to the maximal velocity point within each heartbeat segment.
- **Green vertical lines** denote the segment boundaries defined by adjacent signal peaks.
- **Heartbeat numbers** are displayed above each segment for easy identification and cross-referencing with the CSV output.

(2) **Tabular Output (CSV):** The pipeline also generates a structured CSV file containing the following columns for each heartbeat:

- **Heartbeat number**: Sequential index of the cardiac cycle.
- **Y Position (pixels)**: Raw vertical pixel coordinate of the peak point.
- **Converted PSV**: Calibrated peak systolic velocity (in physical units).

In addition to per-beat data, summary statistics—including the mean and standard deviation of converted PSV values—are appended to the end of the CSV file to support aggregate analysis.

## Part 6: Algorithm
